# Occurrence and identification of lungworms in Iranian wild boars

**DOI:** 10.1007/s00436-024-08313-y

**Published:** 2024-08-08

**Authors:** Younes Ghahvei, Maryam Khani, Shahrzad Azizi, Hosein Khovand, Shokoofeh Shamsi

**Affiliations:** 1 Department of Pathobiology, Faculty of Veterinary Medicine, Shahid Bahonar University of Kerman, Kerman, Iran; 2 Faculty of Veterinary Medicine, Baft Islamic Azad University, Kerman, Iran; 3 School of Agricultural, Environmental and Veterinary Sciences, Gulbali Institute, Charles Sturt University, Wagga Wagga, Australia

**Keywords:** *Metastrongylus pudendotectus*, *Metastrongylus salmi*, Scanning electron microscopy, Histopathology, Feral animals, Lungworms, Wildlife

## Abstract

This study represents the first investigation into the occurrence and identification of *Metastrongylus* spp. in wild boars (*Sus scrofa*) in Iran, utilizing both molecular and morphological methods. Thirteen wild boars from Kerman Province were examined, with 92.3% found to be infected with at least one species of *Metastrongylus*. Mixed infections were observed in 38.46% of the animals. Morphological and molecular analyses confirmed the presence of *M. pudendotectus* and *M. salmi*, with prevalence rates of 76.9% and 53.9%, respectively. Histopathological examination revealed transverse and longitudinal sections of *Metastrongylus* parasites within the airways, causing partial to complete obstruction, interstitial pneumonia, and inflammatory responses. The study also highlights the public health significance of these parasites. The higher prevalence observed compared to earlier studies suggests changes in environmental conditions, host dynamics, or agricultural practices as possible factors, warranting further investigation. The findings underscore the need for comprehensive surveillance and control measures to mitigate the risk of zoonotic transmission, particularly in regions with significant wild and domestic swine populations. This study contributes to the understanding of *Metastrongylus* spp. distribution and their pathological impact, emphasizing the ecological importance of wild boars and the necessity for continued monitoring and research to prevent and control infections in both animal and human populations.

## Introduction

The wild boar (*Sus scrofa*) belongs to the family Suidae and is native to extensive regions of Eurasia and North Africa (Oliver 1993). They have a vast distribution in Iran, from bushlands of the Alborz and Zagros mountains in North and West to lower elevations, in areas with dense vegetation, or among wild pistachio trees in relatively open plains (Firouz 2005). Wild boar is an ecologically important species (Pokorny and Jelenko 2013). In Iran, their ecological importance includes providing abundant prey for large carnivores such as leopards. By digging tunnels to find roots, they benefit other animals such as deer to find food. Additionally, pheasants are often seen grazing alongside wild boars. At least 20 different parasitic worm species have been reported in wild boars (Eslami and Farsadhamdi 1992; Humbert and Henry 1989; Jaervis et al. 2007). Among these, *Metastrongylus* spp. are the most prevalent pulmonary worms, infecting the lungs and potentially leading to death, particularly in younger animals. These parasites cause growth retardation, dyspnoea, and bronchopneumonia in the infected pigs (Poglayen et al. 2016). *Metastrongylus* species belong to the family Metastrongylidae. They have a complex life cycle (Taylor et al. 2015). In the adult stage, they reside in the bronchi of boars, where they lay eggs that are excreted in the feces. Earthworms become infected by ingesting these eggs. The third-stage larvae of *Metastrongylus* develop within the earthworm’s digestive system and become infective to boars. When boars consume earthworms harboring these third-stage larvae, the parasitic larvae migrate to the lungs via the mesenteric lymph nodes and right heart, eventually maturing in the bronchi to reach the adult stage (Taylor et al. 2015).

There are several reports of lungworms in Iranian wild boars (Eslami and Farsadhamdi 1992; Mohtasebi et al. 2023; Solaymani-Mohammadi et al. 2003); however, no sequence data are available yet and information about their occurrence in central Iran is limited. The aim of the present study was to determine the specific identity and occurrence of *Metastrongylus* spp. in wild boars in Kerman Province, where there is a lack of knowledge about these parasites.

## Material and methods

This study received animal ethics approval from the Iranian Environmental Protection Organization (IR.UK.VETMED.REC.1399.29). Wild boars were collected from the surrounding areas of the cities of Baft and Jiroft in Kerman Province, southeastern Iran, and inspected for lungworm infections (Table 1). The study was conducted from January 2022 to December 2023. The age of each boar was determined by examining the dental formula and physical appearance. The boars were necropsied, and each organ was closely examined grossly for pathological lesions (Barton et al. 2019; Shamsi et al. 2024). The thoracic and abdominal viscera were separated and placed in individual containers. The surface of the lung lobes was inspected for the presence of grayish nodules and other tissue lesions. The airways were opened with scissors from the trachea to the smallest bronchioles in the caudal lobes, and all visible lungworms were collected. The lungs were then cut into small pieces, pressed in warm water, and examined to recover any invisible worms.

**Table 1:** Details of the examined wild boars and the occurrence of *Metastrongylus* spp. in the present study

Season	No of infected animals, no of examined animals (%)					
	Male	Female	≤ 3 Year age group	> 3 Year age group	Total no of animals	Total no of nematodes
Winter	2, 2 (100)	2, 2 (100)	1, 1 (100)	3, 3 (100)	4, 4 (100)	1872
Spring	0, 1 (0)	2, 2 (100)	1, 1 (100)	2, 2 (100)	2, 3 (67)	637
Summer	2, 2 (100)	1, 1 (100)	0, 1 (0)	2, 2 (100)	3, 3 (100)	978
Autumn	1, 1 (100)	2, 2 (100)	3, 3 (100)	-	3, 3 (100)	925
Total	5, 6 (83)	7, 7 (100)	5, 6 (83)	7, 7 (100)	12, 13 (92)	4412

The collected parasite specimens were transferred to a Petri dish and rinsed with 0.9% physiological saline solution. The worms were then preserved in 70% ethanol and transferred to the parasitology laboratory of the Veterinary Faculty at Shahid Bahonar University of Kerman for morphological examination. In the laboratory, nematode specimens were cleared with lactophenol solution. The identification of the worms at the genus and sometimes species level was conducted using a light microscope (Gassó et al. 2014). All nematodes removed from the lungs were identified based on the sex sac, spicule size, gender, and cuticle protrusions at the end of the female. Finally, nematodes were subjected to further molecular analysis by PCR to determine the species (Brown et al. 2024). A number of nematodes were also selected for scanning electron microscopy (SEM) investigation.

SEM was in accordance with Ghahvei et al. (2024). In brief, the worms were fixed with glutaraldehyde and osmium tetroxide during the primary and secondary fixation processes. The samples were then transferred to the Faculty of Medical Sciences at Ferdowsi University of Mashhad for SEM analysis. The specimens were dehydrated through a graded series of increasing ethanol concentrations and mounted on numbered stubs. The final preparation step involved sputter coating the specimens with gold using an SC7620 fine coater. The stubs were then examined using a LEO1450VP scanning electron microscope operating at 20 kV, and micrographs were prepared.

DNA was extracted using a commercial DNA extraction tissue kit (Roche, Germany) according to the manufacturer’s instructions. A polymerase chain reaction (PCR) amplification was performed to amplify the cox1 gene of the mitochondrial DNA, using the following primer set: Forward: 5′-TTTTTTGGGCATCCTGAGGTTTAT-3′ and Reverse: 5′-TAAAGAAAGAACATAATGAAAATG-3′ (Li et al. 2016). Amplicons were excised from the agarose gel and sent to Pishgam Biotechnology Company for purification and sequencing in both directions, using the same primers as those used for the amplification of the cox1 gene.

Tissue samples from parasitized lungs were taken and fixed in 10% neutral buffered formalin for histopathologic investigation. After fixation, the specimens were processed using standard procedures, and tissue paraffin blocks were prepared. Sections of 5 µm thickness were stained with hematoxylin–eosin and examined under a light microscope.

## Results

**Fig. 1:**
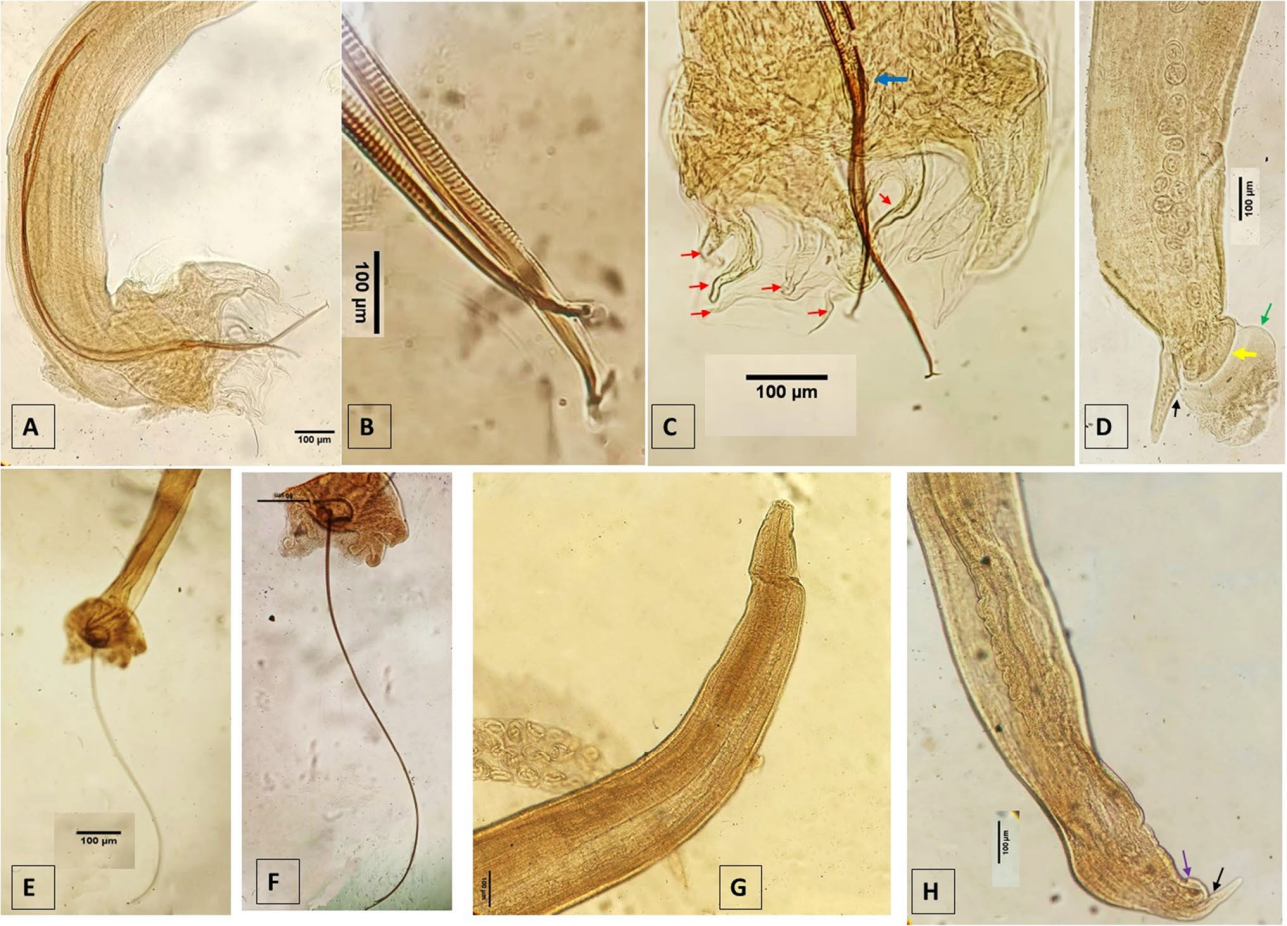
Light microscopy images of parasites found in the present study, including *M. pudendotectus* (**A** to **D**) and *M. slami* (**E** to **H**). **A**, **E**, **F** The posterior end of a male specimen. **B** Tip of the spicule. **C** Gubernaculum (blue arrow) and bursal rays (red arrows, from left to right: ventral ray, anterior ventral ray, anterolateral ray, mid-lateral ray, posterior lateral ray, and external sexual ray). **D** Posterior end of a female, including cuticular dilatation (green arrow), prevulvar cuticular valve (yellow arrow), and anus (black arrow). **G** Anterior end of a female nematode. **H** Posterior end of a female nematode showing vulva (purple arrow) and anus (black arrow)

**Fig. 2:**
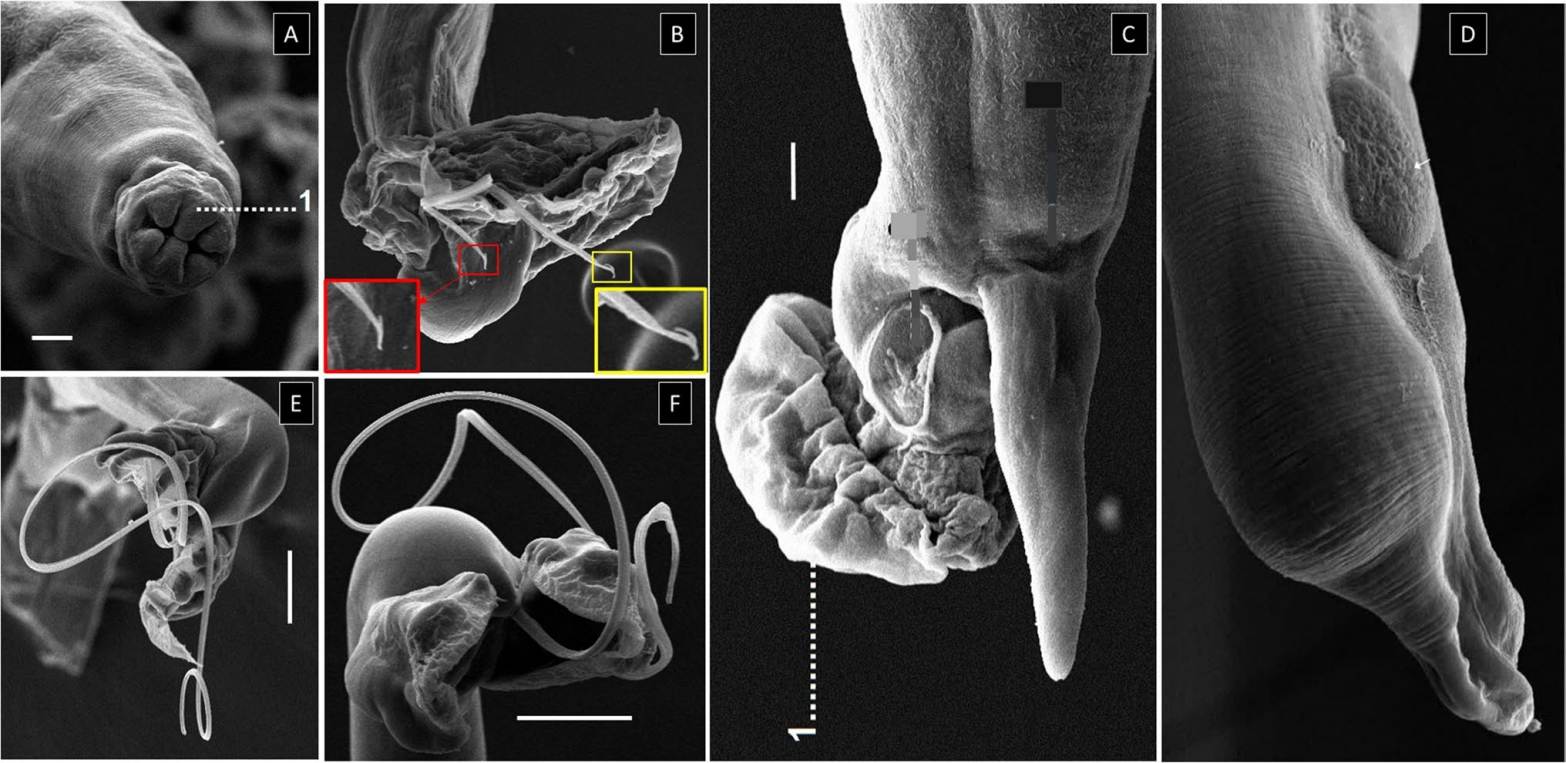
Scanning electron microscopy of **A** anterior end of *M. pudendotectus* showing trilobed labia (1), **B** posterior end of male *M. pudendotectus* (note double barb at the terminal end of the spicules), **C** posterior end of female *M. pudendotectus* (note prevulvar cuticular dilatation (1)), **D** posterior end of female *M. salmi* (note absence of prevulvar cuticular dilatation), **E** and **F** copulatory bursa in *M. pudendotectus* and *M. salmi,* respectively

Morphological identification of the nematodes (Figs. 1 and 2) confirmed them as belonging to the genus *Metastrongylus*, specifically *M. pudendotectus* and *M. salmi*. This identification was further supported by the sequences obtained (GenBank accession number: PP897662) for the former species. Despite several attempts no reliable sequence could be obtained for *M. salmi*. A total of 50 worms from each species were examined, and their morphometric details are provided in Table 2.

**Table 2:** Comparative morphometric data of *M. pudendotectus* and *M. salmi*

	*Metastrongylus pudendotectus*			*Metastrongylus salmi*		
Reference	Gassó et al. (2014)	Panayotova-Pencheva et al. (2019)	Present study	Gassó et al. (2014)	Panayotova-Pencheva et al. (2019)	Present study
Males						
Body length		16.5 (14–21)	16.9 (14.3–19.5)	-	14.3 (11.6–18)	15.9 (12.3–19.5)
Body width at the end of oesophagus	-	108 (90–126)	105 (80–130)	-	98 (87–108)	105 (80–130)
Oesophagus length	-	408 (385–462)	-	-	367 (332–408)	-
Osophagus width	-	64 (58–83)	-	-	60.7 (56–70	-
Spicule length	1380 (1300–1500)	1500 (1300–1700)	1530 (1370–1690)	2120 (1600–2400)	2290 (2150–2450)	2235 (2130–2340)
Gubernaculum length	34 ± 5.55 (27.5–45)	39.7 ± 2.8 (31–46)	-	Not present	26.4 ± 3.1 (23–29)	-
Females						
Body length	-	23 (19–29)	31.5 (22–41)		29.5 (25.2–42.8)	33.5 (26–41)
Body width the end of oesophagus	-	142 (103–174)	-	-	136 (90–179)	-
Oesophagus length	-	454 (423–486)	-	-	454 (407–486)	-
Max. oesophagus width	-	82.5 (68–104)	-	-	77.8 (64–98)	-
Tail length	119 (100–150)	189 (157–217)	199 (167–232)	-	-	88 (71–105)
Length of prevulvar cuticular dilation	262.7 (200–350)	186 (165–207)	186.5 (164–209)	-	-	-
Width of prevulvar cuticular dilation	254.5 (200–330)	160 (139–204)	175.5 (140–211)	-	-	-
Length of prevulvar cuticular valve	165.2 (87.5–200)	147 (125–164)	150 (130–170)	102.92 (75–125)	72.7 (60–93)	76 (63–89)
Width of prevulvar cuticular valve	87.5 (50–137.5)	80 (65–94)	-	58.5 (50–82.5)	61 (47–78)	61 (48–74)
Egg length	56 (50–60)	56.3 (50–67)	58 (51–65)	51.8 (47.5–60)	48.9 (46–58)	50.5 (46–55)
Egg width	43.5 (37.5–55)	35.8 (30–47)	40.5 (32–49)	36.3 (35–37.5)	31.1 (28–36)	32 (29–35)
Distance of vulva from posterior end	-	-	-	-	90 (78–105)	-
Distance anus-tail tip	-	-	-	71 (62.5–92.5)	71 (54–80)	-

A total of 13 wild boars were examined in this study (Table 1), with 12 boars (92.3%) found to be infected with at least one species of *Metastrongylus*. Mixed infections were observed in five (38.46%) animals. The infection rate for *M. pudendotectus* was higher (76.92%) compared to *M. salmi* (53.86%).

The infected lungs exhibited prominent nodular lesions with a gray-pink coloration, ranging from 2 to 5 mm in diameter, visible on the surface of the diaphragm of the posterior lobes. Upon opening the lungs along the bronchi and bronchioles, narrow whitish-yellow nematodes were observed within the airways, associated with mucous-purulent secretions, particularly in the terminal branches of the bronchioles.

**Fig. 3:**
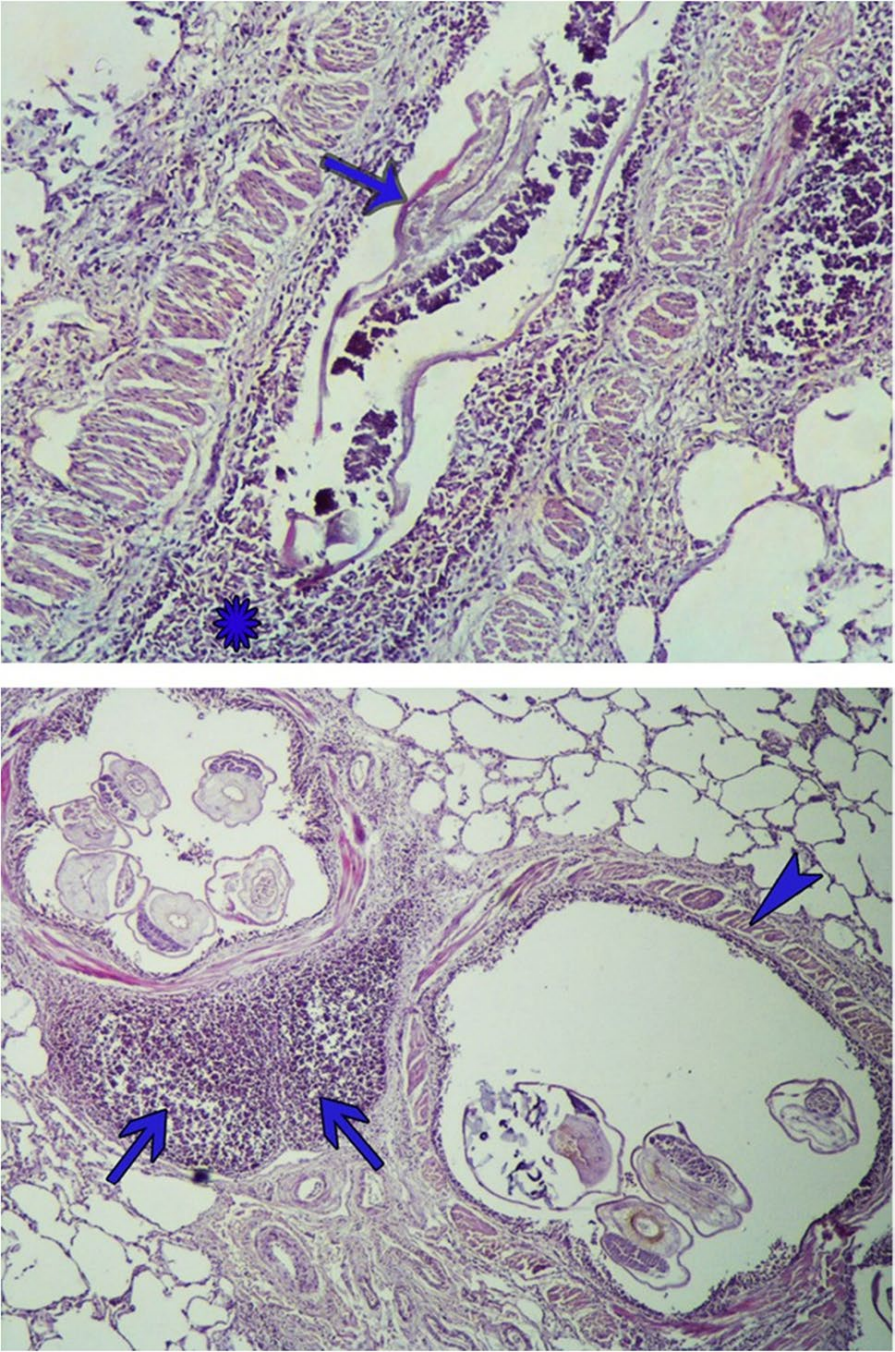
Top: Longitudinal section of *Metastrongylus* nematode (arrow) along with torn and inflammatory epithelial cells (star) inside the bronchiole (hematoxylin–eosin). Bottom: Transverse sections of *Metastrongylus* nematode can be seen inside the bronchioles. Muscle hypertrophy around bronchioles (arrowhead) and BALT hyperplasia (arrows) occurred in the affected lung (hematoxylin–eosin)

Histopathological analysis revealed transverse and longitudinal sections of *Metastrongylus* parasites within the airways, causing partial to complete obstruction in some areas (Fig. 3). The affected lungs demonstrated interstitial pneumonia, characterized by thickening of the alveolar walls. A mild granulomatous response was noted in some regions of the lung parenchyma. The epithelial cells lining the airways were damaged and desquamated. Inflammatory cells, including lymphocytes, neutrophils, and eosinophils, were observed within the lumen of the bronchi and bronchioles. In some areas, airway epithelium became hyperplastic, and inflammatory cells infiltrated the lamina propria. Hypertrophy resulted in thickened smooth muscles around the airways. Hyperplasia of the bronchus-associated lymphoid tissue (BALT) was commonly observed in the infected lungs. Severe hyperemia and hemorrhage were present in certain areas of the pulmonary tissue. Emphysema and atelectasis were other frequently observed lesions.

## Discussion

This study represents the first investigation into the occurrence of *Metastrongylus* spp. in Iran, utilizing both molecular and morphological methods for species identification. While our findings confirm the presence of two species, *M. pudendotectus* and *M. salmi*, it is likely that additional *Metastrongylus* species exist in the country. Both *M. pudendotectus* and *M. salmi* have a global distribution, with reports spanning continents from Oceania to the USA (Beveridge and Emery 2015; Taylor et al. 2015). *Metastrongylus* has worldwide prevalence and comprises six species. Other four species include *M. apri* (also known as *M. elongantus*), *M. confusus*, *M. asymmetricus*, and *M. madagascariensis*. Of these six species, *M. apri, M. pudendotectus*, and *M. salmi* are the most common lungworms in pigs and are usually found in mixed infections (Forrester et al. 1982; Gassó et al. 2014).

Like many other lungworms, *M. pudendotectus* and *M. salmi* can also infect the lower respiratory tract of their hosts (Li et al. 2016). The condition is known as verminous bronchitis or verminous pneumonia, which in young animals may ultimately result in death (Gulbahar et al. 2009; Regassa et al. 2010). These parasites are also known to exacerbate mixed or co-infections with bovine tuberculosis and other viruses (Gassó et al. 2014; Risco et al. 2014).

Due to their worldwide distribution and high prevalence in both wild and domestic swine (Forrester et al. 1982; Nagy et al. 2013; Nosal et al. 2010; Senlik et al. 2011; Solaymani-Mohammadi et al. 2003), it is not surprising that human infections with *Metastrongylus* parasites often occurs (Barton et al. 2023), with cases likely resulting from the accidental ingestion of infected earthworms through contaminated food (Calvopina et al. 2016). These infections underscore the zoonotic potential of *Metastrongylus* spp., emphasizing the need for awareness and preventive measures, particularly in areas where contact with infected wildlife or contaminated soil is common. Although Iran is a country where pigs are not consumed due to being run by a government which enforces Islamic law, the risk posed by lungworms in wild boars remains relevant. The occurrence of human infections due to accidental ingestion of contaminated soil and other animals highlights the importance of proper food handling and sanitation practices to prevent accidental ingestion of parasite-carrying intermediate hosts. Although less commonly infected, other animals such as sheep, cattle, deer, and other ruminants also play a role in the life cycle of these zoonotic parasites and contribute to sustaining their populations. This is particularly relevant in regions like Iran, where these animals are farmed across various provinces and wild boar is widely distributed in agricultural fields and forests of the north, west, and southwest of Iran, with easy access to abundant water and vegetation sources. The presence of *Metastrongylus* spp. in diverse host species in the country underscores the need for comprehensive surveillance and control measures to mitigate the risk of zoonotic transmission.

One interesting finding of this study was the higher prevalence of *Metastrongylus* parasites compared to a previous study conducted in Iran. About two decades ago, Solaymani-Mohammadi et al. (2003) reported the prevalence of *M. pudendotectus* and *M. salmi* to be 16% and 8.3%, respectively. Their study was conducted in Luristan Province, western Iran, which has a more suitable climate for the intermediate hosts of these parasites than the location of the present study. The reason behind this difference could be attributed to various factors such as changes in environmental conditions, host population dynamics, or variations in agricultural practices, which should be investigated in future studies. The global increase in wild boar populations is also expected to change the *Metastrongylus* genus distribution (Acevedo et al. 2014).

The pathological results of the present study are consistent with previous research on pulmonary Metastrongylosis. Rajkhowa and Arya (2015) conducted a histopathological analysis of lungs infected with *M. apri* in pigs, revealing cross-sections of nematodes in the respiratory tracts, obstruction of bronchi and bronchioles, hyperplasia of airway epithelial tissue, BALT hyperplasia, infiltration of mononuclear cells in the alveolar wall, and areas of hyperemia and hemorrhage in the lung parenchyma. Similarly, Panayotova-Pencheva et al. (2019) observed macroscopic lung lesions in the anterior parts of the dorsocaudal lobes infected with *Metastrongylus* species, characterized by irregular gray nodules ranging from 5 to 15 mm in diameter. Their histopathological findings included the presence of parasite sections in the airways, desquamation of bronchial and bronchiolar epithelium, emphysema, and thickening of the alveolar septum due to inflammatory cell infiltration and hyperemia.

We acknowledge that the limited sample size of 13 animals represents a constraint in our study. This limitation should be taken into consideration when interpreting the findings. Despite this, the data gathered provides a preliminary insight into the presence and prevalence of lungworms in wild boar populations in Iran, which can serve as a basis for future research with larger sample sizes.

In conclusion, this study represents the first investigation into the occurrence of *Metastrongylus* spp. in Iran, utilizing both molecular and morphological methods for species identification. Our findings confirmed the presence of two species, *M. pudendotectus* and *M. salmi*, but it is likely that additional species exist in the country. The high prevalence observed, compared to previous studies, suggests evolving environmental and agricultural conditions, which merit further investigation. The global distribution of *Metastrongylus*, with species infecting both wild and domestic swine, underscores the importance of monitoring these parasites due to their potential zoonotic impact. The presence of *Metastrongylus* spp. in diverse host species, including sheep, cattle, and deer, particularly in regions like Iran where these animals are widely farmed, highlights the need for comprehensive surveillance and control measures to mitigate the risk of zoonotic transmission. Although not relevant to Iran due to the Islamic governance of the country, the increasing trend towards free-range pig production systems further contributes to the spread of these parasites, emphasizing the need for vigilant monitoring and control strategies. The pathological findings in our study align with previous research, confirming the significant impact of *Metastrongylus* infections on the respiratory health of their hosts. Future studies should focus on understanding the underlying factors driving the observed differences in prevalence, as well as the potential for human infection, to develop effective mitigation strategies.

## Data Availability

No datasets were generated or analysed during the current study.
